# The impact of autism on the relations between social anxiety, camouflaging, and depression in Taiwanese adolescents

**DOI:** 10.1186/s13034-026-01044-1

**Published:** 2026-02-13

**Authors:** Chun-Hao Liu, Yi-Lung Chen, Wei Ai, Hsing-Chang Ni, Meng-Chuan Lai

**Affiliations:** 1 Department of Psychiatry, Chang Gung Memorial Hospital at Linkou, Taoyuan, Taiwan; 2 Institute of Medical Science, Temerty Faculty of Medicine, University of Toronto, Toronto, ON Canada; 3 Campbell Family Mental Health Research Institute, Centre for Addiction and Mental Health, Toronto, ON Canada; 4 Department of Healthcare Administration, College of Medical and Health Science, Asia University, Taichung, Taiwan; 5 Department of Psychology, Faculty of Arts & Science, University of Toronto, Toronto, ON Canada; 6 College of Medicine, Chang Gung University, Taoyuan, Taiwan; 7 Department of Psychiatry, Temerty Faculty of Medicine, University of Toronto, Toronto, ON Canada; 8 Department of Psychiatry, The Hospital for Sick Children, Toronto, ON Canada; 9 Autism Research Centre, Department of Psychiatry, University of Cambridge, Cambridge, UK; 10 Department of Psychiatry, National Taiwan University Hospital and College of Medicine, Taipei, Taiwan

**Keywords:** Adolescent, Autism, Camouflaging, Depression, Social anxiety, Taiwan

## Abstract

**Background:**

Both camouflaging and social anxiety are adolescence-emerging phenomenon, rooted in social situations, and associated with mental health. The directionality between camouflaging and social anxiety in autistic versus non-autistic adolescents is underexplored. This study aims to investigate the inter-relations between camouflaging, social anxiety, and depression, and the moderating role of autism diagnosis on these inter-relations.

**Methods:**

A total of 205 Taiwanese adolescents (100 autistic, 105 non-autistic) completed self-reported measures, including the Camouflaging Autistic Traits Questionnaire, Chinese version (CAT-Q-Ch), Social Interaction Anxiety Scale, Chinese version (SIAS-C), Generalized Anxiety Disorder 7-item (GAD-7), and Patient Health Questionnaire 9-item (PHQ-9). Network analysis was used to examine the inter-relations among items of the CAT-Q-Ch and SIAS-C. Moderated mediation models, adjusted for generalized anxiety, examined the inter-relations among camouflaging, social anxiety, and depression, and how these associations varied by autism diagnosis.

**Results:**

Autistic adolescents reported significantly higher scores on camouflaging, depression, generalized anxiety, and social anxiety than non-autistic adolescents. A network analysis showed that items from the CAT-Q-Ch formed two communities, and most SIAS-C items formed a third community, supporting the interpretation that camouflaging and social anxiety are related yet distinct constructs. For both autistic and non-autistic adolescents, social anxiety and camouflaging showed bidirectional indirect associations with depression. Critically, having an autism diagnosis significantly weakened the link between camouflaging and depression, as part of the indirect association between social anxiety and depression via camouflaging.

**Conclusion:**

The findings highlight the complex inter-relations among camouflaging, social anxiety, and depression, which may differ between autistic and non-autistic adolescents in Taiwan. Camouflaging may serve different functional roles and have different mental health implications for autistic and non-autistic adolescents. Future research should focus on teasing apart the complex conceptual, measurement, and causal mechanisms underlying the inter-relations between social anxiety and camouflaging in autistic versus non-autistic people.

**Supplementary Information:**

The online version contains supplementary material available at 10.1186/s13034-026-01044-1.

## Background

Autism, a common neurodevelopmental condition, is featured by social communication differences and restricted-repetitive interest, activities and behaviors [1]. Autistic people are vulnerable to mental health challenges, such as depression or anxiety, which may be related to neurobiological and cognitive processing differences, social-contextual adversities, and negative life experiences such as bully victimization [2, 3]. To cope with difficulties in social situations, some autistic people attempt to mask their autistic features using coping strategies, such as camouflaging, which might contribute to poor mental health. Camouflaging refers to strategies that allow one to hide their autistic features and blend into a social situation [4–6]. Qualitative studies found camouflaging to be exhausting, stressful, and causing anxiety and sense of self-alienation in both autistic adults [7–9] and adolescents [10]. Adolescence is a key developmental window to examine given that mental health challenges tend to arise in this period and extend into adulthood [2, 11]. However, how camouflaging mechanistically impacts mental health is still unknown [12].

Social anxiety may play a key role in the relationship between camouflaging and mental health. Social anxiety is a continuum featured by an intense fear in social situations, where one is worried about being negatively evaluated by other people [13, 14]. Social anxiety is common in autistic people, with an estimated lifetime prevalence of 20% in autistic adults [15] and a 3-month point prevalence of 22.9% in autistic adolescents [16]. Social anxiety is associated with poor mental health [17] and commonly co-occurs with depression and generalized anxiety in non-autistic adolescents [18, 19]. Autistic people tend to have greater social anxiety than non-autistic people [20], and may thus experience greater associated mental health challenges.

Social anxiety is also associated with camouflaging in autistic people. On one hand, social anxiety can be an outcome of camouflaging due to one’s social fear of being “found out” having hidden one’s autistic differences [21–24]. On the other hand, social anxiety can also result in camouflaging and burnout, and such relationships seem to be present beyond autistic people [25]. These findings are largely cross-sectional and the directionality cannot be confirmed yet [26]. Theoretically, the social anxiety fueled by unfriendly social environments can drive camouflaging as a defensive response, similar to how “safety behaviors” (e.g., rehearsing one’s speech or performance, used to prevent fearful outcome in social situation) are employed by non-autistic people to mitigate social-evaluative threat [27]. There are currently no longitudinal or causal mechanistic assessments to unravel this potential bidirectionality between camouflaging and social anxiety [12], and how they might differ across neurotypes.

Social anxiety may be experienced differently between autistic and non-autistic people and thus has different impacts to camouflaging and mental health. Traditional theories for social anxiety (e.g., Clark and Wells’s model [28]) might apply differently in autistic versus non-autistic people [20]. Beside social fears of negative evaluation and negative consequences, autistic people’s social fears may be particularly rooted in their social communication differences, contributing differently to camouflaging [29]. Interestingly, recent US general population-based findings suggest that social anxiety may be a less salient driver for camouflaging in those with higher compared to lower autistic traits [30]. Cognitive differences associated with autism, such as cognitive flexibility [31], social attention [32], and uncertainty attunement [33], might all impact how social pressures are interpreted and social anxiety is experienced. The level of autistic traits and/or autism diagnosis might further influence camouflaging and overall mental health [34–36], suggesting a potential moderating role in the relationships among these variables. Camouflaging in autistic people might be less driven by the anxiety that arises from social evaluations but more prominently by tangible threats like physical harm, bullying, and harassment [10, 37]. A recent study additionally showed that there could be aspects of conceptual overlap between social anxiety and camouflaging [38]. It is therefore important to delineate whether the directionality and strength of the relationships among camouflaging, social anxiety, and mental health are different between autistic and non-autistic people.

An important developmental window through which to address these research gaps is adolescence, a critical period for social coping skills development [39, 40] and also for mental health challenges to arise due to increasing social pressures [41]. Social anxiety typically begins in late childhood and early adolescence and persists into later life stages [14, 42, 43]. Although the time when people begin to utilize camouflaging in social situations is still unclear, current evidence indicates that camouflaging might begin to consolidate and become prevalent during adolescence in both autistic and non-autistic people [10, 22, 44–47]. As a result, adolescence is a key time-period to focus on to unpack how camouflaging, social anxiety, and mental health presentations interact with each other.

In sum, both camouflaging and social anxiety are adolescence-emerging phenomena, rooted in social situations, and associated with mental health. These relations can present across neurotypes but show nuanced differences between autistic and non-autistic young people. Accordingly, this study aimed to (1) examine whether social anxiety and camouflaging are associated with each other in relation to depressive symptoms in Taiwanese autistic and non-autistic adolescents, and (2) explore how autism diagnosis moderates these associations. We hypothesized that (1) camouflaging and social anxiety are both associated with depression, indirectly via each other; and (2) these association patterns differ depending on autism diagnosis.

## Methods

### Participants

Adolescents aged 12–18 years (n = 100 autistic and n = 105 non-autistic, respectively) were enrolled. The inclusion criterion of the autistic group was a psychiatrist-confirmed diagnosis of autism spectrum disorder based on the Diagnostic and Statistical Manual of Mental Disorders 5th ed. (DSM-5) or its equivalent DSM-IV criteria. Autistic adolescents were referred by their psychiatrists (including C-HL and H-CN) in the child and adolescent psychiatry outpatient department of the Chang Gung Memorial Hospital at Linkou, Taiwan. If the participant was never evaluated by the study team before, the diagnosis of autism was further confirmed by board-certificated child psychiatrists in the study team (C-HL and H-CN). The inclusion criterion of the non-autistic group was adolescents who were typically developing and without a history of autism. Non-autistic adolescents were enrolled through advertisement posted in Chang Gung Memorial Hospital at Linkou, Taiwan, and through word-of-mouth. Before enrollment, an interview was conducted by the study team to ensure the participants were cognitively capable of understanding the questionnaire items. Caregivers were also involved in this screening procedure to provide clinical history and collateral information to facilitate the confirmation of diagnoses. Adolescents with a history of intellectual disability or learning disorder, or failed to understand the instrument items, were excluded from both groups. Adolescents with a history of developmental delay were also excluded from the non-autistic group. Detailed screening procedure was reported previously [47].

The study was designed to examine the inter-relations among adolescents’ self-reported experiences of camouflaging, social anxiety, and mental health. Intelligence quotient (IQ) was not formally measured in the present study. Importantly, most prior research examining camouflaging and mental health [6, 21, 30, 35, 48–50] similarly did not measure or adjust for IQ and existing evidence found no significant association between camouflaging and IQ in autistic adolescents [51]. Although IQ may correlate with anxiety in the autistic population and remains an important topic for future research [52], previous study of autistic children and adolescents have found the association between camouflaging and internalizing symptoms remains significant even after controlling for IQ [53]. Most importantly, our primary analytic focus is on the interaction effects among self-reported constructs measured within the same context and session; previous methodological work shows that such interaction estimates are more likely to be conservative rather than spuriously inflated when potentially relevant covariates are omitted [54]. Thus, while we acknowledge that the absence of cognitive assessment may reduce precision, the core relational patterns reported are unlikely to be artefactual.

### Measurements

#### Socio-demographic variables

Demographic data including age, sex (assigned at birth), and household income were reported by the participants themselves and their caregivers.

#### Camouflaging autistic traits questionnaire, Chinese version (CAT-Q-Ch)

The CAT-Q-Ch is a 23-item, self-reported questionnaire to quantify camouflaging behavior using a 7-point Likert scale (from 1, strongly disagree to 7, strongly agree). The CAT-Q-Ch was revised from its original 25-item English version Camouflaging Autistic Traits Questionnaire (CAT-Q) [6] and showed excellent internal consistency (Table S1, Supplementary Materials) and test–retest reliability (intraclass correlation = 0.886) based on a psychometric study conducted with autistic and non-autistic adolescents in Taiwan [47]. This dataset also offers initial convergent and divergent validity (Table S2, Supplementary Materials). The original English version has a three-factor structure, including compensation, masking, and assimilation subscales [6]. However, initial evidence shows that the CAT-Q-Ch has a two-factor structure (compensation-masking and assimilation) based on an exploratory factor analysis in adolescents [47].

#### Social interaction anxiety scale, Chinese version (SIAS-C)

The Social Interaction Anxiety Scale (SIAS) is a 20-item, self-reported scale to evaluate anxiety in social interactions in everyday situation [55]. Each item scores from 0 (not at all) to 4 (extremely agree). SIAS has been translated to Chinese and validated in Taiwanese youth [56]. SIAS shows good reliability and validity in the autistic population (Tables S1 and S2, Supplementary Materials) [57].

#### Generalized anxiety disorder 7 item (GAD-7)

The GAD-7 is a 7-item, self-reported questionnaire assessing generalized anxiety symptoms in the past 2 weeks [58]. The Chinese version has good reliability and validity, including in autistic adolescents (Tables S1 and S2, Supplementary Materials) [59].

#### Patient health questionnaire 9 item (PHQ-9)

The PHQ-9 is a 9-item, self-reported instrument evaluating depressive symptoms in the past 2 weeks [60]. The PHQ-9 is widely used for assessing depressive symptoms, with good psychometric properties in both autistic and general populations [61]. The Chinese version is reliable and valid in assessing depressive symptoms in Taiwanese adolescents, including autistic adolescents (Tables S1 and S2, Supplementary Materials) [62].

### Statistical analysis

We examined the differences in all continuous variables between autistic and non-autistic groups using independent samples *t*-tests, and categorical variables using Chi-squared tests. Statistical significance was set at a two-tailed p-value < 0.05. There were no missing data on any the scales. Only seven (3.4%) participants declined to provide their household income, and related analyses were handled by listwise deletion. Network analysis was used to examine the inter-relations among items of the CAT-Q-Ch and SIAS-C. We estimated an undirected and weighted network derived from partial correlations using the EBICglasso (i.e., graphical lasso using extended Bayesian Information Criterion) function in the bootnet R package [63]. We bootstrapped the network model for 1,000 iterations to obtain network-level indices (e.g., dimensionality) and item-level indices (e.g., network loadings). We detected item communities using the fast-greedy algorithm [64] and assessed the modularity scores of the derived community structure. Modularity scores range from -1 to 1 and quantify the density of node edges within detected communities compared to the density expected by chance, with higher values indicating more meaningful community structure.

This study used a cross-sectional design to explore the hypothesized associations among variables. Although mediation models were used to examine the inter-relations among camouflaging, social anxiety, and depression, as well as to test whether these associations differ between autistic and non-autistic adolescents, we refrained from interpreting the identified relationships as implying causal effects and rather considered them as merely correlational in nature (hence the use of “associations” and “indirect associations” when reporting the findings).

**Fig. 1:**
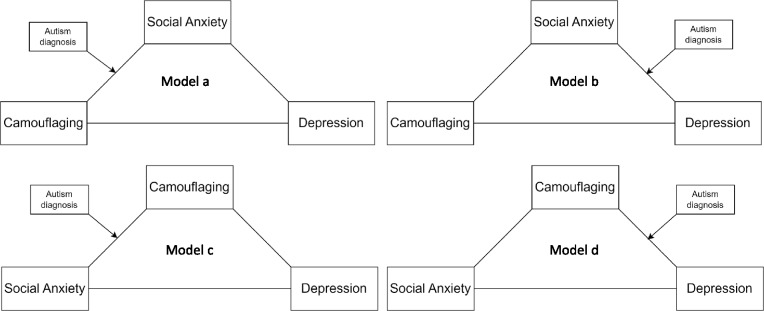
The hypothesized models of the indirect associations between social anxiety and camouflaging and their association with depression

Given the comorbidity between social anxiety and generalized anxiety, concerns have been raised about potential construct overlaps between them [65]. Hence, to control for the potential confounding effect of free-floating generalized anxiety on context-bound social anxiety, we conducted the mediation analysis by controlling for generalized anxiety in the links between social anxiety and camouflaging in the models treating depressive symptoms as the dependent variable (Fig. 1). The “index of moderated mediation” was used to examine the difference in indirect associations (i.e., “indirect effects” as conventionally referred to in mediation models) between the two groups. A bootstrap method with 10,000 iterations was used to construct a 95% bootstrapped confident interval (CI) to examine the indirect and conditional (i.e., moderated) associations. A 95% bootstrapped CI excluding 0 indicates a significant association, and the corresponding effect size (standardized estimate, β) was also reported to reflect the magnitude of the association. Post-hoc statistical power was estimated using a Monte Carlo simulation with 1000 repeats based on the parameter estimates obtained from the fitted mediation model. The mediation modelling was conducted using lavaan [66] and semTools [67] packages, and the Monte Carlo simulation used MASS package [68] in R version 4.4.1 [69].

## Results

**Fig. 2:**
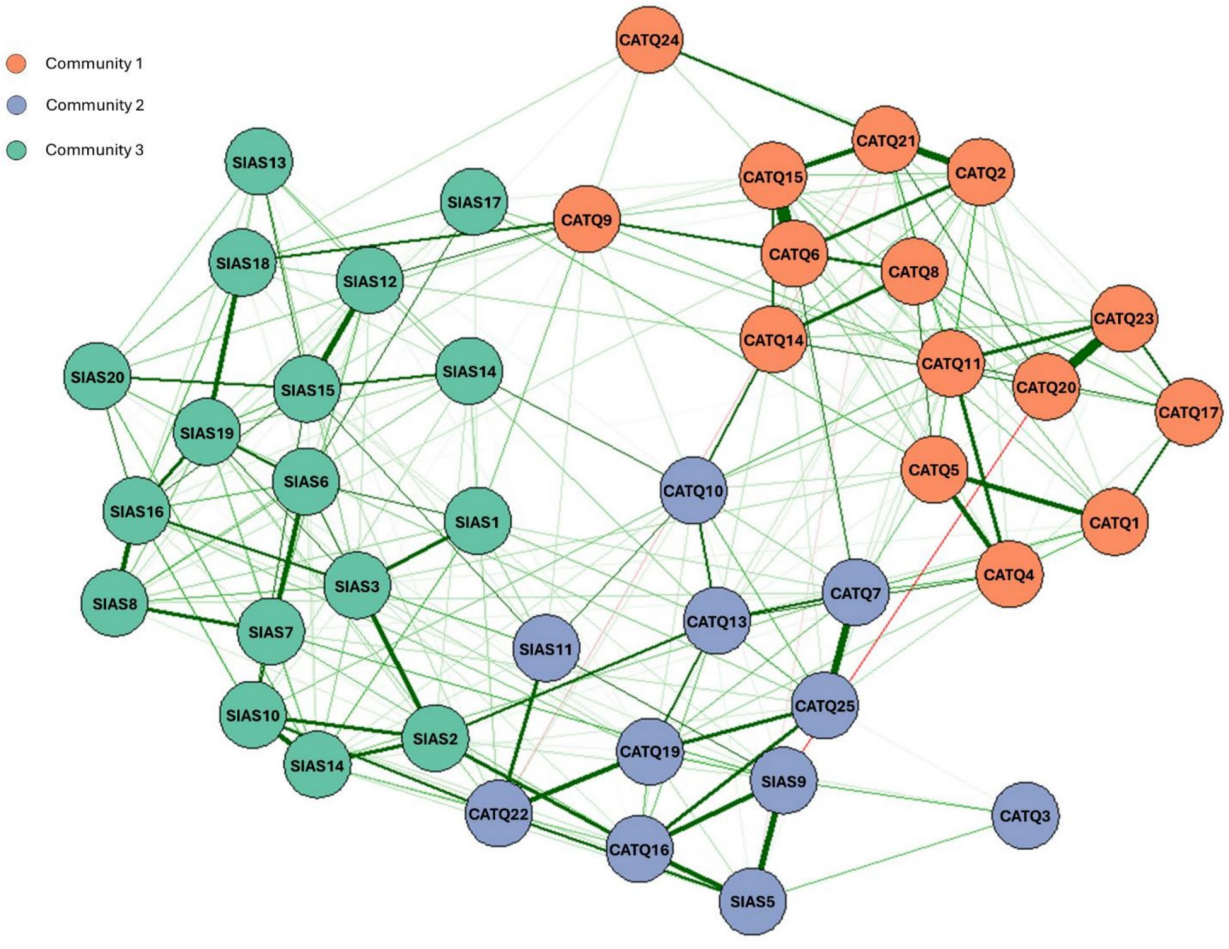
Visualization of network analysis model with a three community structure estimated by fast greedy community detection; Nodes represent individual items in the CAT-Q-Ch and SIAS-C; Node colors indicate community membership; Edges depict regularized partial correlations between items, and edge thickness is proportional to the absolute magnitude of the partial correlation (i.e., stronger correlations reflected by thicker edges); Edge color represents the sign of the correlation (i.e., green represents positive correlations, and red represents negative correlations)

There was no significant difference in sex and age distributions between autistic and non-autistic adolescents. Autistic adolescents self-reported significantly higher total scores than non-autistic adolescents on the CAT-Q-Ch (88.3 ± 23.8 vs. 77.2 ± 21.1, *p* = 0.001, Cohen’s d = 0.49), PHQ-9 (9.0 ± 6.2 vs. 5.0 ± 4.5, *p* < 0.001, d = 0.74), GAD-7 (6.8 ± 5.6 vs. 4.0 ± 4.3, *p* < 0.001, d = 0.56), and SIAS-C (36.9 ± 16.4 vs. 28.4 ± 13.6, *p* < 0.001, d = 0.56) (Table 1). A network analysis on all items in CAT-Q-Ch and SIAS-C showed that items from the CAT-Q-Ch formed two communities, consistent with the two-factor structure derived using an exploratory factor analysis in our previous work [47], and hence were interpreted accordingly. Meanwhile, the vast majority of SIAS-C items (17/20) formed a third, separable community. This overall community partition exhibited a conceptually meaningful structure (modularity = 0.447), supporting the interpretation that camouflaging and social anxiety, as measured by CAT-Q-Ch and SIAS-C respectively, are related yet distinct constructs (Fig. 2). Notably, the three reverse-scored SIAS-C items clustered with the CAT-Q-Ch “assimilation” item community. This finding may in part reflect reverse-wording methodological artifacts. For the SIAS-C specifically, reverse-scored items have shown weaker conceptual convergence and poorer psychometric properties [55]. For the following analyses we kept the previously validated CAT-Q-Ch and SIAS-C item composition as is to maintain the established psychometric properties.

**Table 1:** Differences between the diagnostic groups

	Autistic N = 100	Non-autistic N = 105	p-value	Cohen’s d
Sex (male:female)^a^	71:29	67:38	0.273	
Age (year)	14.9 ± 1.7	14.5 ± 1.8	0.081	0.23
Household income (10,000 NTD/year)^b^	132.5 ± 75.9^c^	123.6 ± 59.1^d^	0.359	0.13
CAT-Q-Ch total score	88.3 ± 23.8	77.2 ± 21.1	**0.001**	0.49
PHQ-9	9.0 ± 6.2	5.0 ± 4.5	** < 0.001**	0.74
GAD-7	6.8 ± 5.6	4.0 ± 4.3	** < 0.001**	0.56
SIAS-C	36.9 ± 16.4	28.4 ± 13.6	** < 0.001**	0.56

CAT-Q-Ch: Camouflaging Autistic Traits Questionnaire, Chinese version; GAD-7: Generalized Anxiety Disorder 7-item; NTD: New Taiwan Dollar; PHQ-9: Patient Health Questionnaire 9-item; SIAS-C: Social Interaction Anxiety Scale, Chinese version

Continuous variable presented as mean ± standard deviation (SD). Bold-faced value represents significance at p < 0.05

^a^Self-reported sex assigned at birth

^b^1 NTD ≒ 0.33 USD

^c^n = 94

^d^n = 104

### Camouflaging and social anxiety showed bidirectional associations in relation to depression

**Fig. 3:**
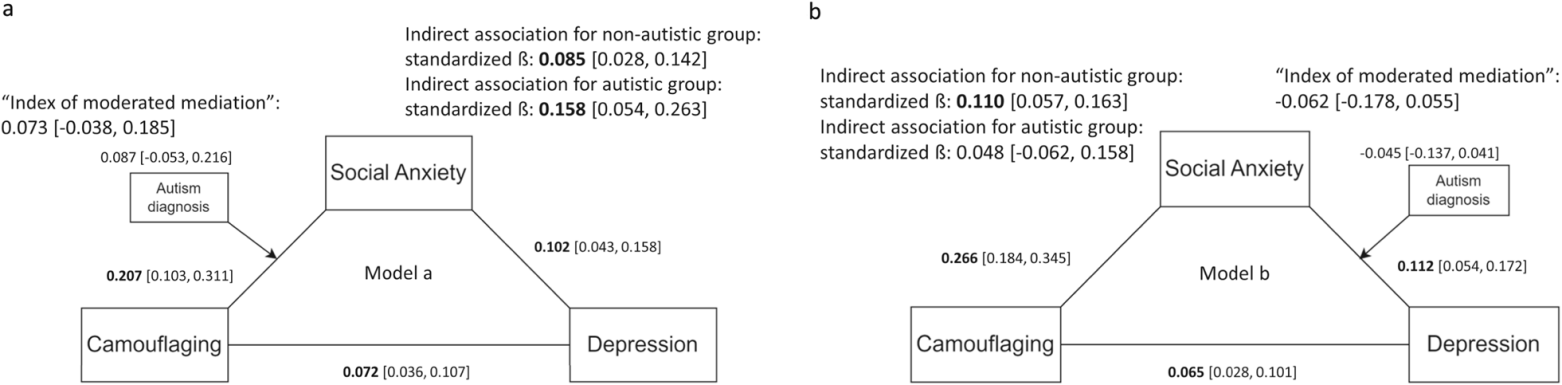
The indirect association between camouflaging and depression via social anxiety, with autism diagnosis modeled as a moderator between camouflaging and social anxiety (**a**) and between social anxiety and depression (**b**)

Controlling for age, sex, and generalized anxiety symptoms, both camouflaging and social anxiety were conditionally associated with depression. When autism diagnosis was modeled as a moderator between camouflaging and social anxiety (model a), the indirect association between camouflaging and depression via social anxiety was significant (standardized ß = 0.085 [0.028, 0.142] for non-autistic, and standardized ß = 0.158 [0.054, 0.263] for autistic adolescents, Fig. 3a). When autism diagnosis was modeled as a moderator between social anxiety and depression (model b), the indirect association between camouflaging and depression via social anxiety was significant only for non-autistic adolescents (standardized ß = 0.110 [0.057, 0.163]), but not for autistic adolescents (standardized ß = 0.048 [− 0.062, 0.158], Fig. 3b).

**Fig. 4:**
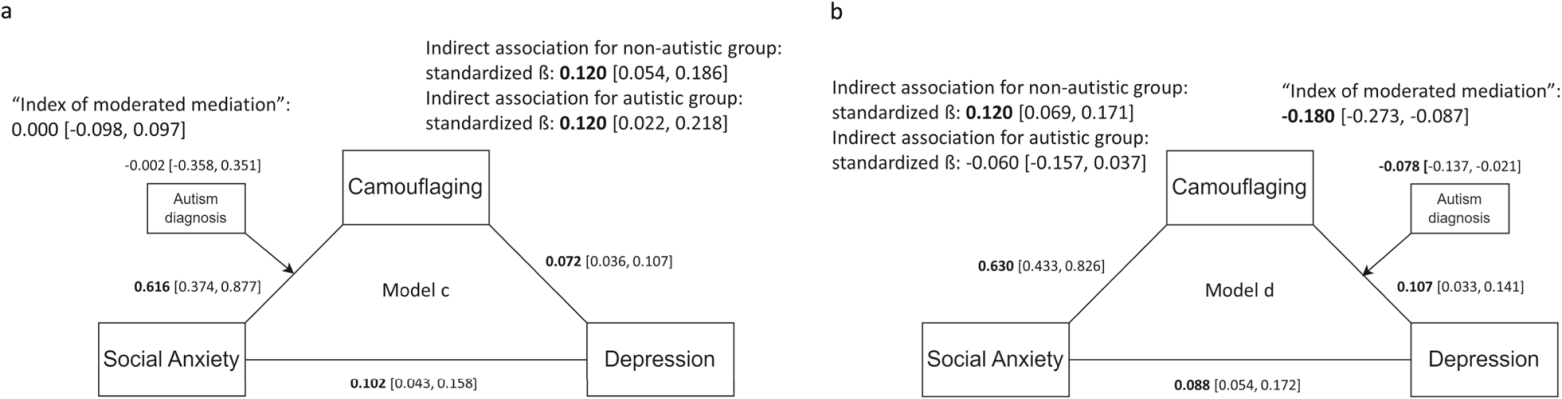
The indirect association between social anxiety and depression via camouflaging, with autism diagnosis modeled as a moderator between social anxiety and camouflaging (**a**) and between camouflaging and depression (**b**)

When autism diagnosis was modeled as a moderator between social anxiety and camouflaging (model c), the indirect association between social anxiety and depression via camouflaging was also significant in both non-autistic (standardized ß = 0.120 [0.054, 0.186]) and autistic adolescents (standardized ß = 0.120 [0.022, 0.218], Fig. 4a). When autism diagnosis was modeled as a moderator between camouflaging and depression (model d), the indirect association between social anxiety and depression via camouflaging was significant for non-autistic adolescents (standardized ß = 0.120 [0.069, 0.171]), but not for autistic adolescents (standardized ß = − 0.060 [− 0.157, 0.037], Fig. 4b).

### The indirect association between social anxiety and depression via camouflaging differed by autism diagnosis

Whether one has an autism diagnosis significantly moderated the indirect association between social anxiety and depression via camouflaging. A significant “index of moderated mediation” (− 0.180, 95% bootstrapped CI [− 0.273, − 0.087], Fig. 4b) means that having an autism diagnosis significantly weakened the indirect association between social anxiety and depression via camouflaging. All other indices were non-significant (Figs. 3a, b, 4a), despite the power was low (Table S3, Supplementary Materials).

### Post-hoc power analysis

A post-hoc power analysis using Monte Carlo simulations given α < 0.05 indicated that our statistical power to detect the indirect association in both autistic and non-autistic groups were all higher than 0.8 (0.812–0.962, see Table S3, Supplementary Materials), indicating adequate power to detect true effects. However, the statistical power for examining “index of moderated mediation” in models a, b, and c were low (0.021 – 0.479), suggesting that the absence of significance should be interpreted with caution. In model d, this index was significant with a post-hoc power of 0.633, indicating a moderate sensitivity to detect true effects.

## Discussion

We found that Taiwanese autistic adolescents reported significantly greater camouflaging and more mental health symptoms than non-autistic adolescents, with medium effect sizes, which was consistent with previous studies [70, 71]. This finding indicates a significant mental health challenge in autistic adolescents in Taiwan that warrants clinical attention. Importantly, social anxiety and camouflaging showed a bidirectional association, such that each was linked to depression through the other, independent of generalized anxiety. The indirect association between social anxiety and depression via camouflaging differed in autistic and non-autistic adolescents, such that having an autism diagnosis weakened the links between camouflaging and depression. Our findings suggest that camouflaging might play different roles contributing to the mental health of autistic versus non-autistic adolescents.

### Bidirectionality between camouflaging and social anxiety: Parallels between camouflaging and safety behaviors, and/or confounding measurements?

We found bidirectional associations between camouflaging and social anxiety in relation to depression, reflecting the intertwined and complex measurement and causal relations. For autistic people who camouflage, they could experience inauthenticity and disconnection and become more self-aware and self-critical of their own social behaviors, which could potentially contribute to increased social anxiety [9, 72]. Camouflaging might in turn become a coping strategy for social anxiety [25]. In addition, despite our network analysis found separable item communities between CAT-Q-Ch and SIAS-C, offering psychometric validity that the two measures tap into separable constructs, camouflaging can phenotypically overlap with safety behaviors such as avoidance (e.g., avoiding being noticed in social situations) and impression management strategies (e.g., trying to fit-in and act “normal”) [73]. Camouflaging can also be conceptualized as a facet of general impression management [4, 12]. Hence, safety behaviors and camouflaging both phenotypically and conceptually overlap with impression management, encompassing efforts to hide one’s struggles and the avoidance of negative evaluation in social situations [20, 50, 74]. Recent research found that camouflaging and safety behaviors could show construct overlaps and are both significantly associated with social anxiety symptoms [50]. Interestingly, we did not find the same measurement and construct overlaps between camouflaging and social anxiety in this Taiwanese adolescent sample. It may be that the shared self-monitoring component (and subsequently increased self-focused attention) in both camouflaging and safety behaviors come to further reinforce anxious thoughts concerning negative social evaluations [20, 50], and this process manifests partially differently in different cultures. Nevertheless, the link between safety behaviors and worsened social anxiety symptoms can be seen in both autistic adolescents [50] and adults [20], echoing the bidirectional relationship between social anxiety and camouflaging we observed.

Another explanation to the bidirectionality is a measurement confound among autistic features, camouflaging, and social anxiety [74]. Social anxiety and autism may share similar avoidant reactions in social situations [75]. Thus, camouflaging as measured by the CAT-Q-Ch may reflect transdiagnostic coping strategies to fit into social expectations and may capture individuals who use camouflaging specifically to cope with social anxiety. When looking deeper into CAT-Q-Ch items (e.g., “In social interactions, I do not pay attention to what my face or body are doing” [reversed]), some may measure the coping strategies used in response to social anxiety or autistic features. To clarify the potential measurement confounds in self-reports versus true underlying relations among autism, social anxiety, camouflaging, and safety behaviors, as well as to further disentangle the overall constructs and fine-grained facets of camouflaging and social anxiety, future studies should employ longitudinal and/or interventional designs, along with comprehensive psychometric evaluation with multi-domain and multi-observer measurements [76].

### Salience of social anxiety and functional roles of camouflaging between autistic and non-autistic adolescents

We found that the associations among social anxiety, camouflaging, and depression were particularly moderated by autism diagnosis, specifically on the links between camouflaging and depression. The indirect association between social anxiety and depression via camouflaging was only seen in non-autistic adolescents, but not in autistic adolescents. Although camouflaging can be used by autistic and non-autistic adolescents and be associated with distress in both cases [44, 47], it may function differently between the two groups, with differential adaptive functions and repercussions. As noted earlier, the measure of camouflaging may have also captured some aspects of safety behaviors and is part of general impression management [77]. For non-autistic adolescents, camouflaging may be primarily used to cope with social anxiety and concerns with social adjustment. For autistic people, camouflaging-safety behaviors can react to both social anxiety as well as experienced social challenges [25, 74, 78]. Particularly in autistic adolescents, camouflaging may be a survival reaction in response to tangible threats like bullying, discrimination, and physical harm [10, 26, 79], on top of social anxiety. This complexity could partly explain why Clark and Wells’ model [28] might work differently in autistic versus non-autistic people. For example, the relationship between safety behaviors and social fear was weaker in autistic compared to non-autistic people, and autism diagnosis itself was a significant predictor of social fear [20]. Because camouflaging-safety behaviors might serve greater adaptive functions for autistic compared to non-autistic adolescents, they may show weaker associations with mental health in autistic than in non-autistic adolescents in Taiwan. Our finding differs from a previous UK-based study showing that people with greater autistic traits tend to engage in more camouflaging and exhibit poorer mental health [80]. In a collectivist-prone culture like the Taiwanese society, a certain level of camouflaging might uniquely serve an adaptive role, helping autistic individuals better fit into society and thus could be, in some cases, associated with more positive mental health.

Also, this noted pattern appears consistent with general population-based findings in adults, such that social anxiety is a more salient driver of camouflaging for people with lower than higher autistic traits [30]. Moreover, the relationship between safety behaviors and social anxiety also seems weaker in autistic than non-autistic people [20]. Alternatively, autism-specific features such as differences in social attention [81], or their automaticity and habituation of camouflaging [5, 82], might attenuate the role of camouflaging on the association between social anxiety and depressive symptoms. With extended measurements, future investigations can clarify these potential mechanisms and explore how social coping strategies can be employed in adaptive ways such that the mental health costs can be reduced in both autistic and non-autistic adolescents.

### Limitations

Several limitations should be considered. First, this study uses a cross-sectional design and cannot confirm the temporal or causal relations among variables despite the use of mediation models. For example, we cannot rule out the reverse causality that depressive symptoms result in higher levels of camouflaging or social anxiety. Also, shared variance or omitted variables can account for the observed bidirectional associations. Second, the statistical power of “index of moderated mediation” was low, indicating that the absence of indirect associations moderated by autism diagnosis should be interpreted with caution, and replication in larger samples is warranted. Third, although the questionnaires used in this study (e.g., PHQ-9, GAD-7, SIAS-C) are widely utilized, they are originally developed for mostly neurotypical populations. Also, measurement invariance between autistic and non-autistic adolescents has not been fully evaluated. Therefore, the interpretation of findings in autistic individuals should be made with caution. Future studies will benefit from incorporating assessment tools specifically developed or validated for autistic populations. Fourth, we did not collect data on participants’ IQ, educational information, concurrent mental health diagnoses, or intensity of autistic characteristics. The absence of such factors may limit the interpretability of our results. For example, it is plausible that well-masking autistic adolescents might have been included in the non-autistic group. How these factors may influence the associations among social anxiety, camouflaging, and depression warrants further investigation. Finally, this study focused on the relations between camouflaging and social anxiety, without measuring other possible confounding factors, such as social skills, cognitive functions, and experienced oppression and adversity (e.g., bullying, stigmatization), which can influence both camouflaging and social anxiety, as well as overall mental health.

### Clinical implications

Our findings imply that camouflaging plays distinct roles in Taiwanese autistic and non-autistic adolescents, with partially different implications for mental health. For autistic adolescents, camouflaging may facilitate smoother social navigation but is often associated with psychological costs, highlighting the importance of supportive and accepting environments to reduce the pressure to camouflage. For non-autistic adolescents, camouflaging appears more strongly linked to maladaptive coping with social anxiety, underscoring the need for interventions that promote healthier strategies.

## Conclusion

We found that social anxiety and camouflaging exhibit bidirectional associations concerning their relationships with depression in Taiwanese autistic and non-autistic adolescents. Moreover, camouflaging as arising from social anxiety may have different impacts on depressive symptoms between autistic and non-autistic adolescents. These findings should be interpreted with considerations of potential conceptual overlaps between camouflaging and safety behaviors as well as measurement limitations of self-report questionnaires for the constructs regarding camouflaging and social anxiety. Longitudinal and interventional studies, with broader and more robust measurements, are necessary to clarify the directionality of associations among camouflaging, safety behaviors, social anxiety, and their relations with mental health outcomes, and how these relations may differ between autistic and non-autistic young people.

## Supplementary Information

Additional file 1.

## Data Availability

The datasets used and/or analyzed during the current study are available from the corresponding author on reasonable request.

## Abbreviations

CAT-Q-Ch: Camouflaging Autistic Traits Questionnaire, Chinese version
GAD-7: Generalized Anxiety Disorder 7-item
PHQ-9: Patient Health Questionnaire 9-item
SIAS-C: Social Interaction Anxiety Scale, Chinese version
