# Supplementary material for: The impact of autism on the relations between social anxiety, camouflaging, and depression in Taiwanese adolescents
Source: Child Adolesc Psychiatry Ment Health. 2026 Feb 13;20:40. doi: 10.1186/s13034-026-01044-1 (PMC13005511; doi:10.1186/s13034-026-01044-1)
Supplement: Supplementary file 1 — Additional file 1. [file 13034_2026_1044_MOESM1_ESM.docx]

**Supplementary Materials**

**Table S1.** Cronbach’s α for the total scores and subscales of CAT-Q-Ch, PHQ-9, GAD-7, and SIAS-C

|  | Autistic group | Non-autistic group |
| --- | --- | --- |
| CAT-Q-Ch Total score | 0.908 | 0.902 |
| CAT-Q-Ch Compensation-Masking subscale | 0.918 | 0.890 |
| CAT-Q-Ch Assimilation subscale | 0.812 | 0.800 |
| PHQ-9 | 0.853 | 0.833 |
| GAD-7 | 0.919 | 0.892 |
| SIAS-C | 0.920 | 0.900 |

CAT-Q-Ch: *Camouflaging Autistic Traits Questionnaire, Chinese version*; GAD-7: *Generalized Anxiety Disorder-7 item;* PHQ-9: *Patient Health Questionnaire-9 item;* SIAS-C: *Social Interaction Anxiety Scale, Chinese version*

**Table S2.** The Pearson’s correlation among CAT-Q-Ch and theoretically related (PHQ-9, GAD-7, SIAS-C) and unrelated constructs (household income)

|  | CAT-Q-Ch | PHQ-9 | GAD-7 | SIAS-C |
| --- | --- | --- | --- | --- |
| PHQ-9 | 0.465  p<0.001 |  |  |  |
| GAD-7 | 0.481  p<0.001 | 0.812  p<0.001 |  |  |
| SIAS-C | 0.562  p<0.001 | 0.467  p<0.001 | 0.519  p<0.001 |  |
| Household income ^a^ | 0.106  p=0.136 | 0.095  p=0.182 | 0.019  p=0.785 | 0.088  p=0.216 |

CAT-Q-Ch: *Camouflaging Autistic Traits Questionnaire, Chinese version*; GAD-7: *Generalized Anxiety Disorder-7 item;* PHQ-9: *Patient Health Questionnaire-9 item;* SIAS-C: *Social Interaction Anxiety Scale, Chinese version*

^a^ n=198

**Table S3.** Monte-Carlo simulation estimating the post-hoc statistical power of the “indirect effect” and “index of moderated mediation” of the models

|  | Standardized coefficients | 95% confident interval | | Power | |
| --- | --- | --- | --- | --- | --- |
| Model a | | | | | |
| Indirect effect – non-autistic group | **0.085** | 0.028 | 0.142 | | 0.936 |
| Indirect effect – autistic group | **0.158** | 0.054 | 0.263 | | 0.938 |
| Index of moderated mediation | 0.073 | -0.038 | 0.185 | | 0.021 |
| Model b | | | | | |
| Indirect effect – non-autistic group | **0.110** | 0.057 | 0.163 | | 0.860 |
| Indirect effect – autistic group | 0.048 | -0.062 | 0.158 | | 0.812 |
| Index of moderated mediation | -0.062 | -0.178 | 0.055 | | 0.479 |
| Model c | | | | | |
| Indirect effect – non-autistic group | **0.120** | 0.054 | 0.186 | | 0.962 |
| Indirect effect – autistic group | **0.120** | 0.022 | 0.218 | | 0.956 |
| Index of moderated mediation | 0.000 | -0.098 | 0.097 | | 0.035 |
| Model d | | | | | |
| Indirect effect – non-autistic group | **0.120** | 0.069 | 0.171 | | 0.906 |
| Indirect effect – autistic group | -0.060 | -0.157 | 0.037 | | 0.892 |
| Index of moderated mediation | **-0.180** | -0.273 | -0.087 | | 0.633 |
